# Complement Attack against *Aspergillus* and Corresponding Evasion Mechanisms

**DOI:** 10.1155/2012/463794

**Published:** 2012-08-09

**Authors:** Cornelia Speth, Günter Rambach

**Affiliations:** Division of Hygiene and Medical Microbiology, Innsbruck Medical University, Fritz-Pregl-Straße 3, 6020 Innsbruck, Austria

## Abstract

Invasive aspergillosis shows a high mortality rate particularly in immunocompromised patients. Perpetually increasing numbers of affected patients highlight the importance of a clearer understanding of interactions between innate immunity and fungi. Innate immunity is considered to be the most significant host defence against invasive fungal infections. Complement represents a crucial part of this first line defence and comprises direct effects against invading pathogens as well as bridging functions to other parts of the immune network. However, despite the potency of complement to attack foreign pathogens, the prevalence of invasive fungal infections is increasing. Two possible reasons may explain that phenomenon: First, complement activation might be insufficient for an effective antifungal defence in risk patients (due to, e.g., low complement levels, poor recognition of fungal surface, or missing interplay with other immune elements in immunocompromised patients). On the other hand, fungi may have developed evasion strategies to avoid recognition and/or eradication by complement. In this review, we summarize the most important interactions between *Aspergillus* and the complement system. We describe the various ways of complement activation by *Aspergillus* and the antifungal effects of the system, and also show proven and probable mechanisms of *Aspergillus* for complement evasion.

## 1. *Aspergillus* Evokes Invasive Infections in Immunocompromised Individuals

*Aspergillus *species are ascomycetes that are classified in the form subdivision Deuteromycotina, as many of them do not show a sexual reproductive phase [1]. Generally, they are common ubiquitous saprophytes in soil and on dead organic substrates. Being classic opportunistic pathogens, invasive infections by *Aspergillus *species almost exclusively develop in immunocompromised patients, while localized infections and allergic bronchopulmonary aspergillosis occur in individuals without immunosuppression. Generally, the species *Aspergillus fumigatus* represents the most common inducer of invasive and allergic manifestations, followed by *A. terreus, A. flavus, *and *A. niger* [1, 2].

Invasive aspergillosis (IA) considerably contributes to the morbidity and mortality among immunocompromised individuals, including patients with haematological malignancies, recipients of haematological stem cell and solid organ transplants, AIDS patients, and patients treated with immunosuppressive regimens due to autoimmune diseases [3]. The most important single risk factor is prolonged and profound neutropenia (<500 neutrophils/*μ*L for more than 10 days) [1, 4–6]. Over the last decades, invasive fungal infections, particularly aspergillosis, have become more frequent due to a higher number of immunocompromised patients (new chemotherapy regimens, increasing number of solid organ transplant recipients, and immunosuppressive regimens) and extended survival time in HIV patients (HAART therapy) [1, 7–9].

On the side of the pathogen, several characteristics and various putative virulence factors that may facilitate the infection have been described for *A. fumigatus*. It differs from nonpathogenic species by its growth at 37°C; furthermore, it is rapidly growing and has very small conidiospores (3–5 *μ*m) [1]. These include melanin and a hydrophobic protein-coat layer on the surface of conidia that may help to protect them against recognition, ingestion and/or elimination by complement and phagocytes [10–14]. Various proteases that may help to pass tissue barriers and to degrade proteins of the immune response are secreted by the fungus [15–17], and mycotoxins like gliotoxin might also contribute to undermine the host defence [18–20]. 

The most important path of *Aspergillus* infections is via inhalation of the conidia into the respiratory tract. As conidia of pathogenic *Aspergillus* species are very small, they can be inhaled deeply into the lung and even into the pulmonary alveoli [1]. In immunocompetent individuals, conidia are effectively phagocytosed and eliminated by alveolar macrophages and infiltrating neutrophils [12, 21, 22], but in the case of immunologic deficits, they are able to germinate and to penetrate the lung tissue, thus causing an invasive pulmonary aspergillosis.

Infections of the lung are the by far most frequent type of IA. By penetration of blood vessels, *Aspergillus *can disseminate and invade other organs, including the heart, the liver, and the central nervous system (CNS). Cerebral aspergillosis occurs in 10%–20% of all cases of IA and thus is the most common extrapulmonary form [1]. Neuropathologic features include hemorrhagic infarcts and/or necrosis, vascular thrombosis, meningitis, granuloma, and formation of solitary as well as multiple abscesses [6, 23–25].

According to the Division of Bacterial and Mycotic Diseases (DBMD), the incidence of aspergillosis is 1-2 per 100,000 per year. Incidence rates of IA in high-risk populations depend on the respective group and rise up to 24% in patients with prolonged and profound neutropenia [4]. Furthermore, IA is the most expensive opportunistic infection in immunosuppressed patients, with annual treating costs in Europe of approximately € 200 million. In-hospital stays complicated by IA cause additional costs of € 75,000 per patient. 

Despite antimycotic therapy and surgical interventions, the fatality of IA is high and depends on the degree of immunosuppression and on the affected organs. Without treatment, the mortality is nearly 100%, while under treatment the overall case-fatality rate is nearby 60% and rises to more than 90% in cases of CNS aspergillosis [1, 6, 26].

## 2. Complement: An Innate and Sophisticated Antimicrobial Defence Mechanism

### 2.1. Three Activation Pathways Mediate Recognition of Foreign Structures

Complement consists of approximately 30 fluid-phase and membrane-bound proteins that cooperate to form the cascade. Regulatory factors control and modulate its activity, and cellular receptors mediate the interaction between complement factors and immune cells. Representing a potent component of the innate host defence and an interface to adaptive immunity, it displays a multitude of physiological activities and functions. The most outstanding roles are the direct and indirect defence against infections, the stimulation and regulation of B- and T-cell response, and the disposal of debris [27–31]. Hepatocytes are the main producers of complement factors; however, several other cell types participate in the synthesis.

**Figure 1:**
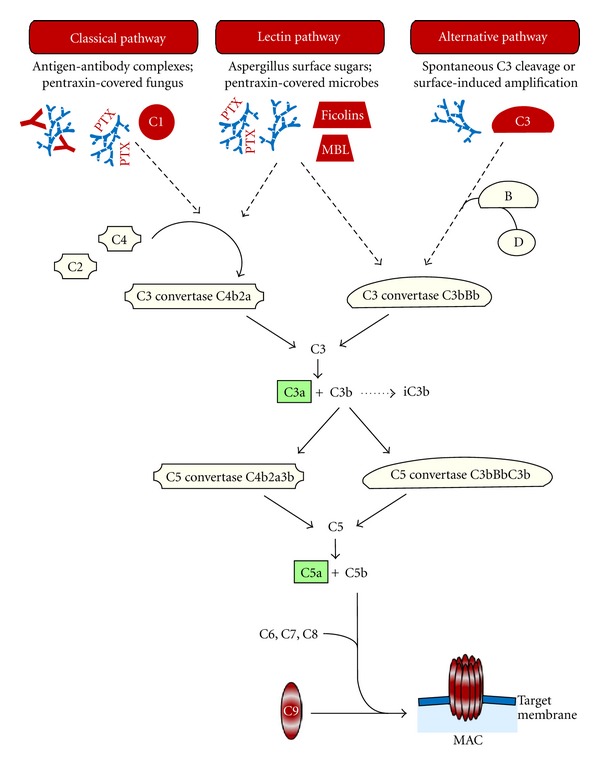
Scheme of complement activation pathways induced by *Aspergillus* and the subsequent course of the complement cascade. For details, see text. PTX: pentraxin; MAC: membrane attack complex.

Activation of the complement system is triggered by a multiplicity of “danger signals,” such as pathogen-associated molecular patterns (PAMPs), antigen/antibody complexes, and the presence of transformed cells, apoptotic cells, or cell debris. Three different activation pathways start the complement cascade, all of them resulting in the cleavage of the central complement factor C3 by proteolytic enzyme complexes (C3 convertases), and subsequently leading to the common terminal pathway (Figure 1) [2].

In the classical pathway, binding of complement factor C1q to immunoglobulin class G or M (IgG, IgM) of antigen-antibody complexes represents the initial step [27]. Alternatively, the globular heads of C1q can interact with microbial surfaces that had been covered by pentraxins, a class of soluble pattern recognition molecules [32]. The thereby induced conformational changes of C1q subsequently activate the associated proteases C1r and C1s, which cleave the factors C4 and C2. The resulting fragments form the C3 convertase C4b2a [27]. 

In the lectin pathway, foreign carbohydrate molecules on the surface of pathogens are recognized by mannose-binding lectin (MBL) or the related ficolins [33]. MBL-associated serine proteases (MASPs) cleave C4 and C2, and the fragments build up the C3 convertase C4b2a, which is identically equal to the one of the classical pathway. Ficolin-2 can also interact with pentraxin-covered microbes, thus starting the lectin pathway in an alternative manner [34]. Interestingly, MBL was recently described to support C3 cleavage by a C2 bypass mechanism [35], which results in activation of the alternative pathway.

The alternative pathway is triggered via activating foreign surfaces and creates an amplification loop by spontaneous reaction of C3 with H_2_O (C3(H_2_O)); alternatively, C3b generated by the other pathways represents the starting trigger. Surface-bound C3b associates with factor B, which is then cleaved by the plasma serine protease factor D. These steps result in the formation of the C3 convertase C3bBb [27, 36]. 

### 2.2. All Activation Pathways End in a Common Terminal Pathway

Proteolytic cleavage of C3 by one of the C3 convertases is the common and central step of all three activation pathways. This split generates the fragments C3a and C3b, which are two important components that mediate a multitude of complement functions (see below). The product C3b associates with the C3 convertases, thus forming the C5 convertases, which cleave factor C5 into C5a and C5b. This step initiates a chain of assembly processes of the proteins C6, C7, C8, and C9. The bound and polymerized C9 units create the terminal complement complex (TCC) that can form a pore in the target lipid bilayer, called membrane attack complex (MAC). Targeted cells, bacteria and viruses die or are inactivated by efficient disruption of the membrane integrity [31, 37].

### 2.3. Other Antimicrobial Functions of Complement Activation Products

Beneath the MAC formation and direct pathogen destruction, complement displays several additional antimicrobial mechanisms aiming to neutralize invading microbes and to restore body homeostasis. Surface-bound C3b undergoes internal cleavage steps; the derived products iC3b, C3d, and further, coat and label the pathogens for phagocytosis (opsonization). Effector cells with specific membrane-bound complement receptors (CRs) recognize the opsonizing complement fragments, ingest the labeled pathogens, and eliminate them. Furthermore, the interaction of the opsonized particles with CR-bearing immune cells results in their activation and their increased proliferation [27]. The receptor CR3, a heterodimer of CD11b and CD18, is regarded to be the most important mediator for complement-driven phagocytosis. Being expressed on phagocytes like dendritic cells, neutrophils, macrophages, and microglia, it interacts with iC3b on the pathogen [27, 38].

The complement receptors CR3 and CR4 allow adhesion of cells to cells of the same and other cell types, respectively, (homotypic and heterotypic adhesion). Immune cells can bind via these receptors to their ligands on endothelium of the blood vessels, a prerequisite for penetration through the vessel wall into the tissue and migration to the site of infection and inflammation [27].

Surface-bound iC3b can be further cleaved proteolytically to generate the opsonizing fragment C3d. Binding of C3d-opsonised pathogens to the corresponding complement receptor CR2 (CD35) on B cells induces cross-linkage with the B cell receptor complex, a process that lowers the threshold for B cell activation by the specific antigen by several orders of magnitude [39]. Consequently, the production of antibodies is stimulated via this complement-dependent process [39].

Cleavage of C3 and C5 generate the potent anaphylatoxins C3a and C5a, respectively, which exert several biological functions by binding to their corresponding cellular receptors C3aR, C5aR (CD88), and C5L2. They provoke chemotactic attraction of immune cells to the site of infection and an increase of vascular permeability [40, 41]. Furthermore, C3a and C5a trigger an efficient proinflammatory response by stimulating cytokine synthesis and secretion [40, 41]. Various cell types harboring the corresponding anaphylatoxin receptors on their surface react on ligand binding with cell activation, stimulation of cell specific signaling pathways, or of oxidative burst [27, 42]. 

### 2.4. Regulator Molecules Strictly Control the Course of the Complement Cascade

The complement cascade needs a tight control to prevent host damage by cell/tissue lysis and excessive inflammation. A variety of both soluble and membrane-bound regulators can influence all steps of the complement cascade, with the C3/C5 convertases as main control targets [27, 37, 43–45]. Under normal conditions, these regulators should protect all body cells against auto-attack by the complement system. 

The serine protease factor I cleaves both C4b and C3b and is thereby supported by various cofactor molecules. C4 binding protein (C4 bp) and factor H (FH) are fluid-phase proteins that enable the cleavage of C4b and C3b, respectively, Moreover, the membrane-anchored molecules complement receptor 1 (CR1, CD35) and membrane cofactor protein (MCP, CD46) support the degradation of both C3b and C4b. In addition, FH and C4 bp accelerate the decay of assembled C3 convertase; CR1 affects both C3 and C5 convertases. Decay accelerating factor (DAF, CD55) is another notable membrane-bound regulator that efficiently prevents the assembly and promotes the disintegration of both C3 and C5 convertases [27, 43–45]. 

In the terminal pathway, the membrane-anchored CD59 (protectin) binds to C8 in the C5b-8 complex and thus inhibits further incorporation and polymerization of C9 units to form the MAC [27, 43–45].

### 2.5. Complement in Infectious Diseases: Beneficial and Detrimental Effects

The potency of complement represents a valuable tool to attack invading pathogens and to defend the host against penetration and dissemination. One particular advantage of complement lies in the fact that activation can start within seconds after contact with the microbe and ends with a multifaceted spectrum of antimicrobial reactions. However, the fact that microbial infections occur in a considerable proportion, already implicates that the pathogens have developed appropriate counterstrategies to avoid elimination, thus starting a vicious circle of reaction and counterreaction.

Furthermore, the antimicrobial effector mechanisms of the complement system might also harbour harmful consequences for the affected host. As known from several infectious and noninfectious diseases, chronic or exceeding complement-mediated inflammation can also contribute to tissue damage in the course of these diseases. Putative mechanisms for such complement-induced tissue damage may include a fulminant inflammatory reaction and opsonization of surrounding “bystander” cells with subsequent lysis. 

## 3. Complement Activation by *Aspergillus*

*Aspergillus* conidia and hyphae activate the complement system via all three pathways [46–48] (Figure 1).

Initiation of the complement cascade by resting conidia is mediated predominantly by the alternative pathway. However, when the conidia begin to swell and transform into hyphae, there is a progressive involvement of the classical pathway [46]. These differences in the activation pathways are reflected by different kinetics; the slowest initiation is seen with resting conidia [46].

Furthermore, MBL as a pattern recognition molecule of the lectin pathway is able to bind to carbohydrate structures on the surface of *Aspergillus* and promotes complement activation via the lectin pathway, which results in the deposition of C4 [48]. As mentioned above, MBL can support C3 cleavage by a C2 bypass mechanism after contact with *A. fumigatus *conidia, resulting in activation of the alternative pathway and avoiding formation of the classical pathway C3 convertase [35]. This mechanism is not restricted to *A. fumigatus,* but can also take place in the presence of *A. terreus, A. niger,* and *A. flavus.*

MBL generally seems to be a molecule of high significance for innate defence against a range of pathogens. In several studies, it was shown to bind to various sugars on the surfaces of viruses, bacteria, yeasts, fungi, and protozoa [48–51]. Further evidence for the crucial role of MBL arises from findings in patients with chronic necrotizing pulmonary aspergillosis and mouse models of pulmonary aspergillosis [52, 53]; these facts suggest MBL as a promising molecule for prophylaxis and therapeutical treatment (see below). 

A further mechanism for complement activation driven by *Aspergillus* involves the interaction with the pattern recognition molecule pentraxin-3 (PTX-3). When *A. fumigatus* is opsonized with PTX-3, the complement cascade can be activated either by interaction between PTX-3 and C1q via the classical pathway [32], or by interaction between PTX-3 and ficolin-2 via the lectin pathway [34].

After seroconversion, anti-*Aspergillus* antibodies in the serum can trigger the start of the classical complement pathway [46].

## 4. Complement Exerts Antimicrobial Functions against Invasive *Aspergillus *Infections 

The thesis that complement represents a central tool in antifungal host defence is supported by several findings. Complement deficiency is correlated with enhanced susceptibility to a disseminated infection by *A. fumigatus* [54]. Furthermore, recognition by the complement system and activation of the cascade seems to interfere with efficient dissemination in the host. This conclusion is strongly indicated by the fact that the level of complement deposition on different *Aspergillus* species correlates inversely with their pathogenicity: highly virulent species like *A. fumigatus* and *A. flavus* bind less C3 on their surface than nonpathogenic species like *A. glaucus* or *A. nidulans* [55].

The antimicrobial potency of the complement cascade appears to be independent from direct killing via formation of a MAC; presumably, the thick fungal cell wall block the formation of a pore by the C9 polymers and the subsequent lysis of the cells [56]. Other complement-derived effector molecules are more effective to cope with aspergillosis. Opsonization of the fungal surface with C3-derived fragments are presumably the most relevant complement-associated weapon, stimulating efficient phagocytosis or release of damaging compounds, oxidative burst and killing by monocytes, bronchoalveolar macrophages, and polymorphonuclear cells [46, 47, 57]. The capacity to opsonize pathogens and to exert antifungal effects strictly depends on the available complement levels in the respective compartment of the body. The complement concentrations in the central nervous system (CNS) are low and thus only allow a rather weak deposition [58]. Consequently, the complement amounts in the cerebrospinal fluid are unable to induce a significant oxidative burst in immune cells and to result in reduced fungal viability, thus making the CNS a highly vulnerable organ. However, the brain cells react to the fungal presence with an upregulation of complement synthesis to enable better opsonization and therefore a more efficient clearance of the fungus [58]. 

A second complement effector mechanism involves molecules of the terminal complement pathway. Mice deficient in complement factor C5 can exert the early opsonization processes with C3 fragments, but are unable to fulfil the complete cascade. When infected with *A. fumigatus,* these mice show decreased resistance and lower 50% lethal conidia dosage for a disseminated infection [59, 60]. Since this enhanced susceptibility is unlikely to be due to absent MAC formation in the fungal cell membrane, it might be supposed that the inability to form the anaphylatoxin C5a could be the relevant deficit in these mice [59, 60]. C5a exerts a wide range of proinflammatory effects; by binding to its receptor C5aR, C5a recruits inflammatory cells to the site of infection, enhances cellular adhesion, and stimulates oxidative metabolism. In addition, C5a can trigger the release of lysosomal enzymes and of inflammatory mediators such as tumor necrosis factor-alpha (TNF-*α*) and interleukin-6 (IL-6) [61, 62]. Furthermore, a higher susceptibility to aspergillosis in C5-deficient mice might be attributed to missing TCC. Low doses of this soluble complex were shown to bind to the membrane of a range of cell types, thereby triggering various effects like activation, rescue from apoptosis, and secretion of prostaglandins, which are important regulators of the immune response [63–67]. 

## 5. *Aspergillus* Has Developed a Repertoire of Evasion Mechanisms to Repel Efficient ****Complement Attack

The potency of *Aspergillus* to cope with the complement system and to undermine its mechanisms for elimination determines how successful the fungus can establish an infection [68]. *Aspergillus* has developed a complex repertoire of effector mechanisms for this purpose (Table 1). 

### 5.1. Hiding

Single or multiple abscess formation is a characteristic feature of aspergillosis, particularly in the central nervous system (CNS). The fungal hyphae are found in brain blood, vessels with invasion through vascular walls into adjacent parenchymal tissue. Swelling and inflammation develop in response to the infection. Fungal brain abscesses may arise from these sites of localized parenchymal infection. In this case, white blood cells collect in the affected part of the brain, and fibrous tissue forms around this area, creating a mass. CNS abscesses typically present with headache, focal neurological abnormalities, and/or seizure, which is the consequence of local destruction or compression of adjacent brain tissue [75]. Mature fungal abscesses exhibit a central necrotic area with fungal hyphae, surrounded by a capsule of newly formed fibrous tissue. The formation of abscesses represents a host mechanism to inhibit further spreading of invading pathogens. However, this “sealing off” not only inhibits fungal dissemination, but also forms some kind of protection shields against the complement attack [69]. Immunohistochemical staining revealed that effect: whereas the fibrous surrounding tissue was intensely stained for complement proteins, the central necrotic area contained only minor complement concentrations. No deposition of complement factors on the fungal surface in the abscess was visible, implying that the encapsulation protects the fungus within the abscess from any efficient complement attack [69].

### 5.2. Masking

Putative complement recognition sites on the conidial surface of *A. fumigatus* are optimally masked to minimize the stimulus for complement activation [55]. Experiments aiming to identify the relevant fungal structure indicated that melanin could play a substantial role for masking; for this purpose, knock out mutants lacking enzymes of the melanin biosynthesis pathway were used [10, 11, 76]. Disruption of the gene *alb1*, which encodes a polyketide synthase in the synthesis of melanin, results in increased opsonization of the conidia with C3 and in a better ingestion by human neutrophils [11]. Deposition of pigments on the conidial surface might mask the C3 binding sites, and disruption of the alb1 gene might expose these sites and thus allow enhanced C3 binding [11]. A mouse model confirms this function of *alb1 *in fungal pathogenesis, since the alb1-deficient mutant of *A. fumigatus* turned out to be less virulent than the wild-type fungus [11, 76]. Inactivation of the gene for the pigmentation protein *arp1* similarly increased the deposition of C3 on conidia [10]. However, the detailed mechanism how melanin decreases complement deposition remains unclear. This pigment seems to be a central element of *Aspergillus* against the host defence, as it is also involved in scavenging reactive oxygen species (ROS) and inhibits the acidification of phagolysosomes of alveolar macrophages, monocyte-derived macrophages, and human neutrophil granulocytes after ingestion of conidia [12, 13, 77].

### 5.3. Acquisition of Complement Inhibitors

As mentioned above, the activity of the complement cascade is strictly limited by several fluid-phase inhibitors. Immunofluorescence analysis, adsorption assays and flow cytometry studies showed that *Aspergillus* acquires FH, factor H-like protein 1 (FHL-1), factor H-related protein 1 (FHR-1), and C4 bp from the host [70, 71]. FHL-1 is a splicing product of the FH gene, and FHR-1 is a related protein belonging to the FH family. Bound to the conidial surface, FH maintained its regulatory activity and could act as a cofactor for the factor I-mediated cleavage of C3b [71]. As a consequence of covering the fungal surface with these complement inhibitors, all three pathways might be downmodulated. The attachment molecules on *Aspergillus* are not yet known, whereas the corresponding binding regions within FH were recently described [71]. One of them was identified within N-terminal short consensus repeats (SCRs) 1 to 7 and a second one within C-terminal SCR 20 [71].

### 5.4. Production of Complement Inhibitors

*A. fumigatus* not only acquires complement inhibitors from the host, but also produces and releases its own soluble factor that inhibits complement activation and opsonization of the fungus [72, 73]. This complement inhibitor (CI), which is also synthesized by *A. flavus*, selectively abolishes activation of the alternative pathway and interferes with C3b-dependent phagocytosis and killing [72]. The exact chemical composition of CI is as yet unknown; it contains 15% protein and 5% polysaccharide. Further biochemical characterization suggested that phospholipids of *A. fumigatus* contribute to its functional activity [73]. Recent own results raise the possibility that this described CI or a closely related activity also contributes to the pathogenesis in cerebral aspergillosis, since immunohistochemical studies show deposition of C1q and C4, but not of C3, on the fungal hyphae in the CNS [69]. 

### 5.5. Degradation of Complement Proteins

Studies by Sturtevant revealed the synthesis of a proteolytic enzyme that is able to degrade C3 ([47], reviewed in: [74]). This is confirmed by own experiments showing complement-degrading proteolysis in the supernatant of *Aspergillus* when grown in cerebrospinal fluid (CSF) [17]. The fungus-induced degradation of complement in CSF evoked a drastic reduction of the opsonization of the fungal hyphae. In parallel, the phagocytosis of the conidia by neutrophils and microglia decreased significantly. The fungal serine protease Alp1 might participate in complement degradation and thus be partly responsible for the complement evasion [15, 17]. To date, degradation of C1q, C3, C4, C5, MBL, and factor D were shown [15, 17].

## 6. Therapeutic Approaches: What Can We Learn from the Conflict between *Aspergillus* and Complement? 

The high morbidity and lethality of invasive aspergillosis strongly demands for an expansion of the current treatment options. The antimycotic therapy might be completed by new approaches aiming to strengthen the host immune response against the fungus. Putative approaches could be to increase the available complement concentrations or to improve the efficiency of complement attack by undermining the fungal evasion strategies. Appropriate strategies that target the complement system may aim to the following. 

### 6.1. Increasing the Available Complement Protein Levels

Our own studies about cerebral aspergillosis showed a clear correlation between the complement levels in the CSF and the capacity of CSF to opsonize fungal hyphae and designate them for phagocytic killing [58]. A therapeutic increase of MBL concentrations in invasive aspergillosis might be an appropriate approach, since patients with chronic necrotizing pulmonary aspergillosis show more frequently MBL haplotypes that encode for low levels of the protein than healthy control persons [52]. Further support comes from a murine model of invasive pulmonary aspergillosis: those mice with externally administered recombinant MBL reveal better survival, compared to untreated animals [53]. Detailed studies confirmed that MBL-treated mice show a significant increase in the levels of the proinflammatory cytokines TNF-*α* and IL-1, together with a marked decrease of anti-inflammatory IL-10 and of fungal hyphae in the lung [53].

### 6.2. Undermining *Aspergillus-*Driven Complement Evasion Strategies

The development of therapeutic approaches interfering with the fungal complement evasion is highly speculative. Blocking of the fungal surface pigments by specific antibodies or peptides might be a hypothetical approach that could help to expose the C3 binding sites and thus improve complement deposition and ingestion of conidia by phagocytes. Another approach might target the acquisition of the negative complement regulators FH, FHL-1, and C4 bp to the fungal surface. For *Candida albicans*, some molecules that bind C4b and FH have recently been identified [78, 79], while the attachment sites on *Aspergillus* are still unknown but might include related molecules. Blocking antibodies, designed peptides or other inhibitors against these complement regulator binding molecules might help to make the fungus more vulnerable towards complement attack. A similar approach might be developed for the *Aspergillus*-derived complement inhibitor described by Washburn [72, 73]. 

Our own results open the possibility to neutralize the fungal protease(s) that is/are secreted by *Aspergillus* to degrade complement proteins [17]. Two different strategies were tested by first *in vitro* experiments: the neutralization of the protease by specific inhibitors or interference with the production of this proteolytic enzyme. In our studies, we could prevent the complement degrading activity by serine protease inhibitors [17]. However, a therapeutically used protease inhibitor must be highly specific, since a general block of serine proteases might be fatal for the host. Alternatively, our experiments exhibited that the secretion of complement-degrading enzymes strictly depends on the availability of nitrogen sources [17]. Thus, the supply of amino acids in the infected host might downmodulate the secretion of the relevant fungal protease that cleaves the complement factors of the host.

## 7. Summary and Conclusion

Despite new antifungal drugs and improved medical treatment, invasive aspergillosis remains a dangerous threat for immunocompromised patients, as the innate immune defence is the most crucial weapon against this infection.

The complement system is of particular importance, as it harbours multiple effects against infectious diseases, bridges the elements of the human defence network by a multitude of factors, and helps to preserve the homeostasis of the body. The presence of fungal pathogens is detected by different pattern recognition molecules; three pathways guarantee the activation of the complement cascade by resting, swollen, and germinating conidia as well as by hyphae of *Aspergillus*.

Direct lysis of fungal cells by the membrane attach complex (MAC) appears to be of minor importance for the antifungal defence. Presumably, attraction and activation of immune cells (monocytes, pulmonary macrophages, and polymorphonuclear neutrophils) are the most essential mechanisms. Anaphylatoxins (C3a, C5a) chemotactically recruit immune cells to the site of the infection and induce further inflammatory reactions. Opsonization of conidia and hyphae with complement fragments like C3b and iC3b mediate phagocytosis, oxidative burst, and release of damaging compounds by binding to corresponding receptors on immune cells.

However, highly virulent *Aspergillus* species have evolved mechanisms to evade the attack by complement. They hide from recognition, acquire complement regulatory molecules from the host, and secrete proteases to degrade complement factors. 

The multifaceted interactions between complement and *Aspergillus* represent promising approaches for future therapeutic strategies that may help to improve the outcome of invasive aspergillosis.

## Figures and Tables

**Table 1:** Overview on how *Aspergillus* copes with the complement system.

Mode of action	Reference	Effects
Hiding	[69]	Fungal abscess forms a shield that protects against complement attack by inhibiting diffusion of complement factors to the hyphae
Masking	[10, 11]	Fungal surface compounds (pigments) avoid recognition by complement factors and reduce deposition of C3 on conidia
Acquisition of complement inhibitors	[70, 71]	Host-derived FH, FHL-1, FHR-1, and C4bp are bound on the fungal surface and downmodulate complement activation
Production of complement inhibitors	[69, 72, 73]	Synthesis of a fungal complement inhibitor (CI) that interferes with the complement cascade
Proteolytic degradation of complement proteins	[15, 17, 47, 74]	Fungal protease(s) degrade(s) complement proteins, thus avoiding formation of antimicrobial effector molecules
